# Predicting inhospital admission at the emergency department: a systematic review

**DOI:** 10.1136/emermed-2020-210902

**Published:** 2021-10-28

**Authors:** Anniek Brink, Jelmer Alsma, Lodewijk AAM van Attekum, Wichor M Bramer, Robert Zietse, Hester Lingsma, Stephanie CE Schuit

**Affiliations:** 1 Department of Internal Medicine, Erasmus MC, Rotterdam, The Netherlands; 2 Medical Library, Erasmus MC, Rotterdam, The Netherlands; 3 Public Health, Erasmus MC, Rotterdam, The Netherlands

**Keywords:** triage, crowding, acute care, emergency department, research, epidemiology

## Abstract

**Background:**

ED crowding has potential detrimental consequences for both patient care and staff. Advancing disposition can reduce crowding. This may be achieved by using prediction models for admission. This systematic review aims to present an overview of prediction models for admission at the ED. Furthermore, we aimed to identify the best prediction tool based on its performance, validation, calibration and clinical usability.

**Methods:**

We included observational studies published in Embase.com, Medline Ovid, Cochrane CENTRAL, Web of Science Core Collection or Google scholar, in which admission models were developed or validated in a general medical population in European EDs including the UK. We used the Critical Appraisal and Data Extraction for Systematic Reviews of Prediction Modelling Studies (CHARMS) checklist to assess quality of model development. Model performance was presented as discrimination and calibration. The search was performed on 11 October 2020.

**Results:**

In total, 18 539 articles were identified. We included 11 studies, describing 16 different models, comprising the development of 9 models and 12 external validations of 11 models. The risk of bias of the development studies was considered low to medium. Discrimination, as represented by the area under the curve ranged from 0.630 to 0.878. Calibration was assessed in seven models and was strong. The best performing models are the models of Lucke *et al* and Cameron *et al*. These models combine clinical applicability, by inclusion of readily available parameters, and appropriate discrimination, calibration and validation.

**Conclusion:**

None of the models are yet implemented in EDs. Further research is needed to assess the applicability and implementation of the best performing models in the ED.

**Systematic review registration number:**

PROSPERO CRD42017057975.

**Key messages**

**What is already known on this subject**

- Several admission prediction tools have been developed with the intention to shorten length of stay at the ED in an attempt to reduce crowding. Implementation of a tool into every day practice has not yet occurred, as this can only be done after validation and calibration in the hospital where it is going to be used. No research to evaluate and compare these models has been published yet.

**What this study adds**

- This systematic review is the first to critically appraise these admission prediction tools. Of the 16 models that we reviewed, only few were adequately developed, validated and calibrated.

## Introduction

It is of great importance to provide timely care for patients in the ED. However, sometimes this is compromised by ED crowding, a situation that occurs when there are more patients than available beds in the ED.^1^ ED crowding has direct and indirect detrimental consequences for patient care and ED staff.^2 3^ It leads to an increase in the length of stay (LOS) at the ED, a longer inhospital LOS and an increase in morbidity and mortality.^4–7^ There are several causes proposed for the emergence of ED crowding. Asplin *et al* 8 introduced a conceptual model of ED crowding, visualising the factors associated with crowding. These factors can be divided into input, throughput and output factors. It is thought that mainly output, that is, an inadequate disposition of patients, contributes to crowding, which subsequently leads to limited patient flow at the ED. Especially elderly are at risk for a long LOS and many need to be admitted.^9 10^ Advancing patient disposition may reduce LOS at the ED and thus consequently reduce crowding. The identification of those patients that need admission at ED arrival may help to shorten ED LOS for many patients. Earlier admission (ie, shorter time in the ED) is associated with improved patient outcomes.^11^ Several prediction tools exist to identify patients needing hospital admission. Implementing such a model in clinical practice may alter patient courses and lead to earlier admission.^12^ However, a clear overview of literature concerning admission models has not yet been presented. Therefore, the aim of this systematic review is to give an overview of present knowledge on admission prediction models in a general ED population. The secondary aim was to assess the quality of the developed prediction models. As many studies targeted the older population, we will also provide an overview of prediction models developed for this population.

## Methods

The study was conducted and reported according to the Preferred Reporting Items for Systematic Reviews and Meta-Analyses (PRISMA) guidelines.^13^ We performed a systematic review on prediction models on admission in the ED. The study protocol is registered in the International Prospective Register of Systematic Reviews (PROSPERO) under registration number CRD42017057975.

### Eligibility criteria

We aimed to identify all models developed until 11 October 2020 for a non-trauma ED population or that were applicable to a mixed trauma and non-trauma population. The articles needed to fulfil the following criteria to be considered for inclusion: (1) the prediction tool was developed or validated in an adult population, (2) the prediction model did not have predefined illnesses (eg, pneumonia) or symptoms (eg, tachycardia) as inclusion criteria, and (3) the article described a model rather than only individual predictors. Studies that concerned case reports, reviews or meta-analyses were excluded. Moreover, the search was restricted to articles written in English. The references of eligible studies were analysed to identify additional articles for inclusion. Because of the heterogeneity between ED systems worldwide, we limited our search to prediction models developed or validated in European EDs including the UK.

During our initial assessment of the literature, we found multiple models for the elderly population. Therefore, we also decided to give an overview of this subgroup of models.

### Information sources

The following databases were searched from inception until 11 October 2020: Embase.com, Medline ALL via Ovid, Cochrane CENTRAL Register of Trials via Wiley, Web of Science Core Collection and Google Scholar.

### Search

We used among others the following keywords: prediction, risk, hospital admission, emergency department, model and related synonyms. The queries were developed in Embase.com, and syntax and thesaurus terms were afterwards adjusted for the other databases. The search strategy was created by a biomedical information specialist (WMB). See online supplemental appendix A for the complete syntaxes.

10.1136/emermed-2020-210902.supp1 Supplementary data

### Study selection

Duplicate articles were removed using Endnote for Windows (Thomson Reuters, V.X9) using the method as described by Bramer *et al*.^14^ Two researchers (AB and LAAMvA) independently performed the screening of title and abstracts. Conflicting results were discussed in consensus meetings. After screening the abstracts, the full text of the articles was assessed for eligibility by the same researchers and included or excluded in the systematic review. Any remaining disagreement between the first two researchers was discussed with a third investigator (JA).

### Data collection process & data items

The following data were extracted from every included article: year of publication, author, journal of publication, country of the study, study period, study design, inclusion and exclusion criteria in their study, study population, hospital setting (ie, regional hospital, tertiary care hospital), outcome (ie, number of admissions), model name, parameters within the model, model performance (eg, discrimination and calibration), sample size, derivation and/or validation study, calibration method, handling of missing data and patient characteristics (ie, age, sex). Data were extracted by one investigator (AB) and a random check was performed by a second investigator (JA). This check showed no discrepancies.

### Risk of bias in individual studies

The methodological quality and the risk of bias were assessed with the Critical Appraisal and Data Extraction for Systematic Reviews of Prediction Modelling Studies (CHARMS) checklist, which can be used to describe the reliability, applicability and reproducibility of prediction models.^15^ This checklist is applicable for studies deriving a prediction model. The CHARMS checklist assesses risks for bias in the following areas: participant selection, predictor assessment, outcome assessment, model development and analysis. The results were aggregated into a low, moderate or high risk of bias. The risk of bias was assessed by two investigators (AB and LAAMvA). Discrepancies were discussed with a third investigator (JA).

### Summary measures, data synthesis and analysis

We evaluated the predictive performance of the models described in terms of discrimination and calibration. Discrimination is a measure of how well a model can distinguish the high-risk from the low-risk group for a certain outcome. It is usually presented as an area under the curve (AUC) in which a value closer to 1.0 indicates better distinction.^16^

Calibration reflects the agreement between the expected (ie, predicted) and observed outcome. This can either be assessed by the Hosmer-Lemeshow goodness of fit test, calibration curve, Wilcoxon signed rank test, Schwarz Bayesian Information Criterion or Brier score.^17^ The Hosmer-Lemeshow assesses whether the expected event numbers match the observed event numbers. It provides a χ^2^ statistic and accessory p value. A non-significant p value indicates good calibration. The calibration curve contains a slope and intercept, in which the intercept reflects whether predictions are systematically too low or too high. The calibration slope is a reflection of the predictor effects within the model.^17^ The Wilcoxon signed rank test compares two datasets which are not normally distributed. A significant p value implies that a prediction model performs different in the separate datasets. If a study reported multiple prognostic models or multiple stages of prognostic modelling (eg, development and validation), data extraction was performed separately for each model or stage. We classified prognostic models as separate models when they included a different set of prognostic variables. Models with identical predictors but for different outcomes were considered validation studies.

Patient characteristics were presented as mean with SD, median with IQR or numbers with percentages dependent on the distribution of the data. As a result of the heterogeneity of the patient population and the prediction models, a meta-analysis was not possible.

## Results

### Study selection

**Figure 1:**
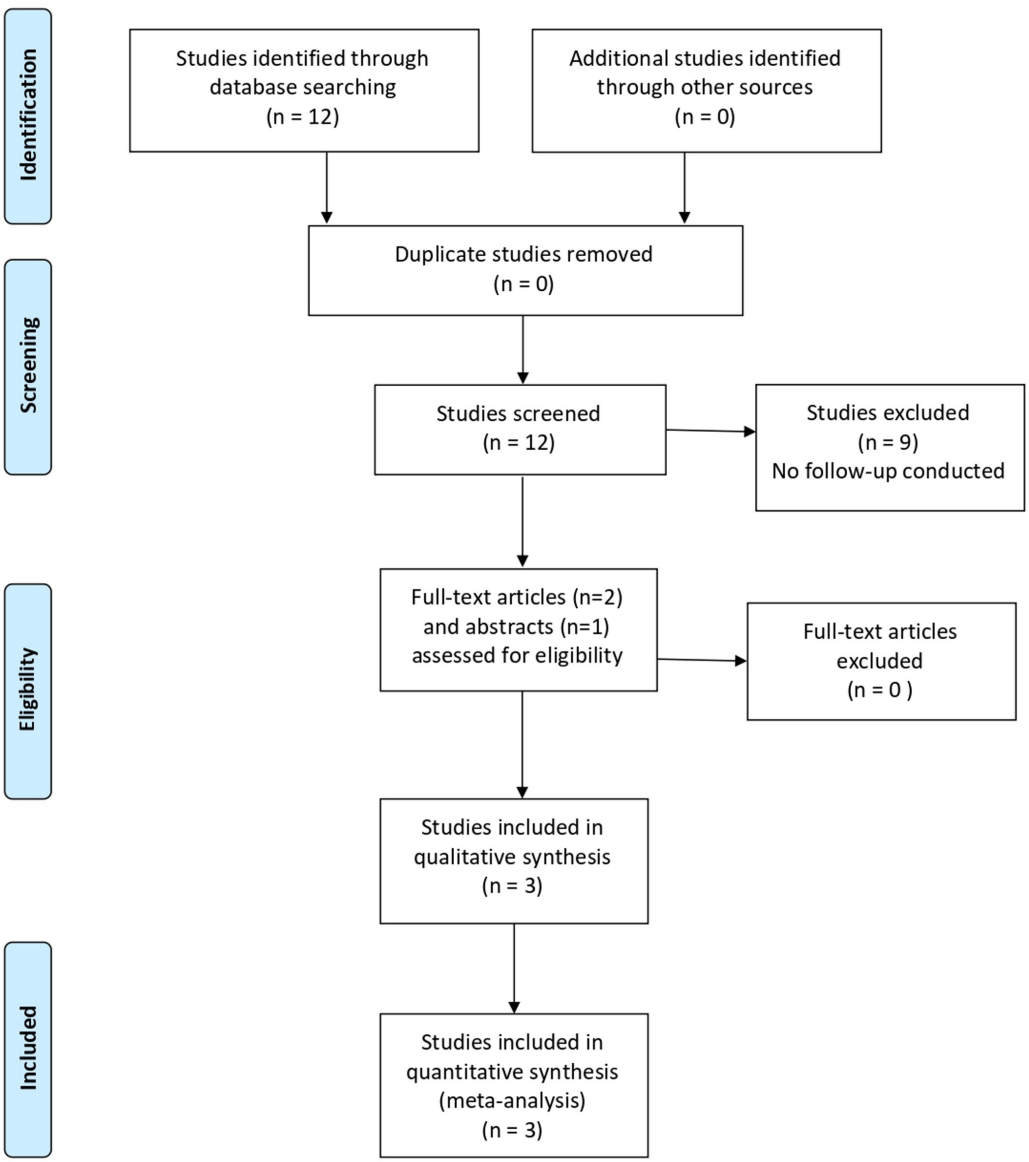
Flowchart for literature search on prediction models for admission.

In the literature search, we detected 18 539 studies, of which 13 017 remained after deduplication. The exclusion of studies based on title and abstract resulted in 104 full text articles eligible for detailed assessment. The main reasons for excluding articles were that the study described was performed in non-European EDs (n=29) or that different outcomes were studied (eg, revisits or LOS) (n=29). Finally, we included 11 articles in this systematic review. Full details of study selection are summarised in figure 1.

### Study characteristics

Study characteristics are summarised in table 1. The 11 studies described 16 different models. Two models were tested in two different studies (Identification of Seniors At Risk (ISAR) and Glasgow Admission Prediction Score (GAPS)).^18–21^ Most models were constructed with logistic regression. Only three models were developed using machine learning.^22 23^

**Table 1:** Characteristics of studies included for systematic review on prediction models for admission for use in the ED (n=11)

Study	Population	Country	Publication year	Journal	Study design	Study period	SC/MC	University hospital/regional hospital	Settings	Study size	Population characteristics
Alam *et al* 28	Adult	The Netherlands	2015	*Resuscitation*	POCS	7 January 2003 to 15 February 2003	SC	University	ED	274	Mean age (SD): 60 (20) ♂(%): 49.0
Brouns *et al* 26	Older	The Netherlands	2019	*BMC Emergency Medicine*	ROCS	September 2011 to August 2012	SC	Regional hospital	ED	20 875	Mean age (SD): 77.0 (7.6) ♂(%): 45.8
Cameron *et al* 19	Adult	Scotland	2014	*Emergency Medicine Journal*	RCSS	21 March 2010 to 20 March 2012	MC	University/regional	ED, AMU, MIU	322 846	NS
Cameron *et al* 18	Adult	Scotland	2016	*Emergency Medicine Journal*	POCS	30 April 2014 to 16 May 2014	SC	University	ED	1829	Mean age (SD): 47.3 (21.0) ♂(%): 48.4
Di Bari *et al* 20	Older	Italy	2012	*Journal of Gerontology*	POCS	January to June 2009	SC	Geriatric hospital	ED	1632	Mean age (SE): 84.0 (5.5) ♂(%): 39.0
Grossmann *et al* 27	Older	Switzerland	2012	*Annals of Emergency Medicine*	POCS	6 April to 27 April 2009	SC	University hospital	ED	519	Median age (IQR): 79 (72–84) ♂(%): 45.9
Kraaijvanger *et al* 24	Adult	The Netherlands	2018	*Emergency Medicine Journal*	POCS	10–16 January 2011, 9–15 May 2011	MC	University/regional	ED	3174	Age: NS ♂(%): 48.8
Lucke *et al* 25	Adult/older	The Netherlands	2017	*Emergency Medicine Journal*	ROCS	January to December 2012	SC	University	ED	21 287	Median age (IQR): DV<70 y: 44.8 (28.8–57.4) DV≥70 y: 78.1 (73.9–83.6) VD <70 y: 44.8 (28.4–58.0) VD≥70 y: 77.9 (73.9–83.0) ♂(%): DV <70 y: 54.6 DV≥70 y: 47.9 VD<70 y: 54.7 DV≥70 y: 50.5
Noel *et al* 22	Adult	France	2018	*European Journal of Emergency Medicine*	PCSS	8 January to 8 February 2016	MC	University/regional	ED	9828	Mean age (SD): DC: 40.8 (22.0), AM: 61.0 (24.0) ♂(%): DC: 51.1 AM: 51.0
Salvi *et al* 21	Older	Italy	2012	*Rejuvenation Research*	POCS	January to June 2009	SC	Geriatric hospital	ED	2057	Mean age (range): 81.7 (65–103) ♂(%): 40.0
Zlotnik *et al* 23	Adult	Spain	2016	*Computers, Informatics, Nursing*	ROCS	January 2011 to December 2012	SC	University	ED	255 668	NS

♂, male; AM, admitted; AMU, acute medical unit; DC, discharged; DV, derivation; MC, multicentre; MIU, minor injury unit; NS, not specified; PCSS, prospective cross-sectional study; POCS, prospective observational cohort study; RCSS, retrospective cross-sectional study; ROCS, retrospective observational cohort study; SC, single centre; VD, validation.

Seven included studies had a prospective design and the majority of the studies were carried out in a single centre (n=8). None of the studies assessed prospectively the performance of the model when implemented in day to day practice.

### Quality assessment

The quality of the studies in which a model was developed (n=5) was assessed using the CHARMS checklist.^19 22–25^ This considered five studies in which nine models were developed. The results of the CHARMS were aggregated into low, medium and high risk for bias (table 2) (online supplemental appendix C).

10.1136/emermed-2020-210902.supp3 Supplementary data

**Table 2:** Risk of bias in the development studies

	Cameron *et al* 19	Kraaijvanger *et al* 24	Lucke *et al* 25	Noel *et al* 22	Zlotnik *et al* 23
Participant selection	L	L	L	L	L
Predictor assessment	L	L	L	M	L
Outcome assessment	L	L	L	L	L
Model development	L	M	L	M	M
Analysis	L	L	L	M	L

L, low risk of bias; M, moderate risk of bias.

The risk of bias is evaluated with the CHARMS checklist, which assesses the domains of participant selection, predictor assessment, outcome assessment, model development and analysis. The results are summarised as low (L) risk of bias, moderate (M) risk of bias or high (H) risk of bias.

The study attrition, referring to the method in which patients were recruited for inclusion, was of good quality in all studies. However, two studies did not describe basic patient characteristics.^19 23^ The outcome was described in all studies. Since the prediction tool had to predict an event in the near future (ie, admission from the ED), loss to follow-up was considered as non-important. Furthermore, the number of patients who were transferred to other hospitals or who left without being seen did not exceed 20%. The number of outcomes in all studies was described and therefore also the number of candidate predictors was satisfying. However, just one study explicitly mentioned that they took into account the number of events per variable to limit overfitting of the model.^25^ In general, the number of events per variable should at least be 10, meaning that if 100 events happened the maximum number of predictors in a model is 10.

All studies included parameters that are easily obtainable during triage. Furthermore, one study provided two models which included the triage nurse prediction on admission.^22^ This is a subjective parameter and therefore difficult to reproduce. However, in the third model by Noel *et al* the triage nurse prediction was not included.

The majority of the models (13/16) were developed using logistic regression, but in three automated computer techniques were used.^22 23^ All studies used age as a categorical variable in the model. However, it is not clearly described whether categorisation of parameters took place before or after inclusion in multivariable analysis.

Description of missing data and handling of missing data were not available for every study,^23 24^ one study excluded patients with missing values^22^ and two studies compensated missing values.^19 25^

External validation is considered to be the best validation method. Two studies performed external validation,^24 25^ while two others used internal validation.^19 23^ One study did not perform validation and was therefore considered a high risk of bias.^22^

Overall, the models of Cameron *et al* 19 and Lucke *et al* 25 were considered to be developed best with an on average low risk on bias in the CHARMS checklist.

#### Participant characteristics

Population size ranged from 274 to 322 846 patients and contribution of male patients ranged from 39.0% to 54.7%. Mean age (SD) ranged from 41 (22) to 84 (5.5) years. Four studies included older ED patients, defined as either ≥65 years^21 26 27^ or≥75 years.^20^ One study compared the older ED population (age≥70 years) with the general adult population.^25^

#### Outcome characteristics

Admission rates varied from 13.6% in adults to 59.4% in the older patient population.

#### Variables included in the scoring systems

The number of parameters ranged from 1 aggregated score (ie, Emergency Severity Index (ESI)) to 13 parameters. We subsequently categorised these parameters into demographics, vital signs, interventions, triage, previous care contacts, chief complaint, drug use, mobility and dependency, ED entrance and professional assessment (table 3). Most scores included demographic information and triage acuity information as predictors for admission.

**Table 3:** Categorisation of parameters in the prediction models

	Model	Demographics	Vital signs	Interventions	Triage	Previous care contacts	Chief complaint	Drug use	Mobility and dependency	ED entrance	Professional assessment
Alam *et al* 28	NEWS		X	X							
Brouns *et al* 26	MTS	X			X						
Cameron *et al* 19 and Cameron *et al* 18	GAPS	X	X	X	X	X				X	
Cameron *et al* 18	VAS										X
Di Bari *et al* 20 and Salvi *et al* 21	ISAR	X				X		X	X		
Di Bari *et al* 20	SC	X				X		X			
Grossmann *et al* 27	ESI				X						
Kraaijvanger *et al* 24	Own model	X			X		X			X	
Lucke *et al* 25	Adult model	X	X	X	X	X	X				X
Lucke *et al* 25	Older patient model	X	X	X	X	X	X				X
Noel *et al* 22	TNP										X
Noel *et al* 22	Own model	X			X		X			X	
Noel *et al* 22	TNP+own model	X			X		X			X	X
Salvi *et al* 21	TRST					X		X	X		X
Zlotnik *et al* 23	Own model LR	X			X		X			X	
Zlotnik *et al* 23	Own model ANN	X			X		X			X	

Online supplemental appendix B.

ANN, Artificial Neural Network; AVPU, Alert, Verbal, Pain, Unresponsive; ESI, Emergency Severity Index; GAPS, Glasgow Admission Prediction Score; GP, general practitioner; ISAR, Identification of Seniors At Risk; LR, logistic regression; MTS, Manchester Triage System; NEWS, National Early Warning Score; SC, Silver Code; TNP, Triage Nurse Prediction; TRST, Triage Risk Screening Tool; VAS, Visual Analogue Scale.

10.1136/emermed-2020-210902.supp2 Supplementary data

### General adult population

We included seven studies that developed or validated a model in the general adult population, aged 18 years and over.^18 19 22–25 28^ In five studies, in total eight prediction models were developed.^19 22–25^

Discrimination in the derivation cohorts of these newly developed models ranged from AUC (95% CI) 0.81 (0.790 to 0.820) to 0.878 (0.876 to 0.879). One study did not provide the derivation AUC, but solely provided the AUCs in the validation population.^24^ Four out of five studies also described validation of their developed models. The remaining two studies tested an existing model in their ED. This consisted of the National Early Warning Score^28^ and the GAPS.^18^ Discrimination in the validation studies ranged from AUC (95% CI) 0.664 (0.599 to 0.728) to 0.876 (0.860 to 0.891). Calibration was described in five studies. Model characteristics are presented in table 4.

**Table 4:** Performance of admission prediction models in the adult population

Study	Model name	Admission, N (%)	Derivation AUC (95% CI)	Calibration method	Calibration derivation	Validation method	Validation AUC (95% CI)	Calibration validation
Alam *et al* 28	NEWS	130 (47.4)				External	t0: 0.664 (0.599 to 0.728) t1: 0.687 (0.620 to 0.754) t2: 0.697 (0.609 to 0.786)	
Cameron *et al* 19	GAPS	NS	0.8778 (0.8764 to 0.8793)	HL GOF test		Split sample	0.8774 (0.8752 to 0.8796)	p=0.524
Cameron *et al* 18	GAPS	745 (40.7)		Wilcoxon Signed Rank test		External	0.876 (0.860 to 0.892)	1.20%
Cameron *et al* 18	VAS	745 (40.7)		Wilcoxon Signed Rank test		External	0.875 (0.859 to 0.891)	9.20%
Kraaijvanger *et al* 24	Own model	400 (31.7)	NS	Calibration plot		External	0.88 (0.85 to 0.90), 0.87 (0.85 to 0.89), 0.76 (0.72 to 0.80)	α: 0.023, β: 0.974 α: 0.05, β: 0.98
Lucke *et al* 25	Own model adults	4044 (23.6)	0.85 (0.84 to 0.86)	Calibration plot, HL GOF test		External	0.86 (0.85 to 0.87)	p>0.05
Noel *et al* 22	TNP	2313 (23.5)	0.815 (0.805 to 0.826)					
Noel *et al* 22	Own model	2313 (23.5)	0.815 (0.805 to 0.825)					
Noel *et al* 22	TNP+own model	2313 (23.5)	0.857 (0.848 to 0.865)					
Zlotnik *et al* 23	Own model LR	34 694 (13.6)	0.8611 (0.8568 to 0.8615)	Calibration plot, HL GOF test	χ^2^= 85.18	Split sample	0.8568 (0.8508 to 0.8583)	χ^2^= 65.32
Zlotnik *et al* 23	Own model ANN	34 694 (13.6)	0.8631 (0.8605 to 0.8656)	Calibration plot, HL GOF test	χ^2^= 16.01	Split sample	0.8575 (0.8540 to 0.8610)	χ^2^= 17.28

Empty cells mean that specific characteristics were not tested.

α, calibration intercept; β, calibration slope; ANN, artificial neural network; AUC, area under the curve; GAPS, Glasgow Admission Prediction Score; HL GOF, Hosmer-Lemeshow goodness of fit; LR, logistic regression; N, number; NEWS, National Early Warning Score; NS, not specified; t, timepoint; TNP, triage nurse prediction; VAS, Visual Analogue Scale.;

### Older ED population

Four studies investigating the older patient population specifically were identified.^20 21 26 27^ In these studies, five different models were described, of which three were older patients specific. These models already existed and were used for predicting either frailty or readmission, but not for primary admission. These models included geriatric parameters, such as cognitive impairment and polypharmacy. Discrimination ranged from AUC (95% CI) 0.63 (0.60 to 0.65) to 0.68 (0.66 to 0.70), which represents poor performance. The other two studies investigated triage systems in older patients.^26 27^ The ESI performed best in predicting admission with an AUC (95% CI) of 0.74 (0.73 to 0.75).^27^ None of the models were calibrated, nor tested in external populations in these articles.

One study compared the older patient population with the general adult population.^25^ This study developed and validated an admission model using temporal validation. The model performed slightly worse in the older ED population, but yielded a good AUC (95% CI) of 0.81 (0.79 to 0.82), which dropped to 0.77 (0.75 to 0.79) after external validation. The positive predictive value and the positive likelihood ratio were higher in the older population. They concluded that further research is needed to investigate the combination of disease severity with frailty to improve prediction of hospital admission in the older patient population. Model characteristics in the older ED population are presented in table 5.

**Table 5:** Performance of admission prediction models in the older population

Study	Model name	Admission, N (%)	Derivation AUC (95% CI)	Validation method	Validation AUC	Calibration method	Calibration
Brouns *et al* 26	MTS	4223 (59.4)		External	0.74 (0.73–0.75)		
Di Bari *et al* 20	ISAR	558 (34)		External	0.65 (0.62–0.68)		
Di Bari *et al* 20	SC	558 (34)		External	0.63 (0.60–0.65)		
Grossmann *et al* 27	ESI	250 (48.8)		External	0.741 (0.734–0.747)		
Lucke *et al* 25	Own model older patients	1817 (43.8)	0.81 (0.79 to 0.82)	External	0.77 (0.75–0.79)	Calibration plot, GOF test	p>0.05
Salvi *et al* 21	ISAR	626 (30)		External	0.68 (0,66–0.70)		
Salvi *et al* 21	TRST	626 (30)		External	0.66 (0.64–0.69)		

Empty cells mean that specific characteristics were not tested.

AUC, area under the curve; ESI, emergency severity index; GOF, goodness of fit; ISAR, identification of seniors at risk; MTS, Manchester triage system; SC, silver code; TRST, triage risk screening tool.

## Discussion

The aim of this systematic review was to find and evaluate prediction models for admission used at the ED. We systematically reviewed 11 papers describing the development or validation of 16 different admission prediction models. Selection of the most appropriate model is based on mainly two qualifications: the model with the lowest probability of overall bias and the highest predictive performances for admission.

Five models reported an AUC over 0.85.^18 19 22 23 25^ The discrimination statistic was highest for the GAPS model.^18 19^

We identified 12 external validations of an admission model. External validation of these models showed substantial variation in performance. This is probably attributable to the fact that some models were tested for a different outcome than they were intended for. Moreover, discrimination may be moderate because ED populations are heterogeneous. Calibration was executed only in 5 of the 11 studies. All models reporting calibration were well-calibrated.^18 19 22 23 25^

Apart from the quality of the model, the model should also be easily applicable. In the ED, it is useful if the parameters used can be obtained directly, are objective and are reproducible. Easily obtainable parameters are predictors that can be retrieved at ED entrance. This will enable immediate use of the prediction model. Several models however use parameters that require (collateral) history, which may limit the utility of the prediction model. This information is often not immediately available. The predictors should also be objective, that is, having a low inter-rater and intra-rater variability. Two studies used the judgement of admission of a healthcare professional as a predictor,^18 22^ which is a subjective predictor.

To allow implementation in clinical practice, models should be easy interpretable or provide applications to enable more complex calculations.

We found that several studies did not report key study details, which made it difficult to judge model utility and make external validation impossible. With the arrival of machine learning in medical prediction research, models have become more complex. The benefit of machine learning is that models improve from experience. However, machine learning limits insight of how the prediction model works and also limits external validation.

### Strengths and limitations

This is to our knowledge the first systematic review on prediction models for admission to the hospital from the ED. Strengths of this study include the comprehensive literature search, selection of articles, standard assessment of the articles and the quality assessment using the CHARMS checklist, which was all performed by two researchers separately. However, also several limitations should be considered. We did limit the inclusion of studies to studies executed in European EDs. We possibly excluded non-European models, which could be applicable to the European ED setting. Even despite only selecting European studies, practice and organisation between countries and even between different EDs in the same country are different. Applicability of a prediction tool is dependent on how the healthcare system is organised. Furthermore, the number of included studies might be reduced by only including studies in English. The general limitation of reporting prediction models for admission in the ED is the heterogeneity in ED patients, which is due to epidemiological differences in the populations. This makes it difficult to compare prediction models and to combine these studies in a meta-analysis.

### Future directions

None of the studies described implementation, and to our knowledge, none of the models are currently implemented in the ED as a prediction tool for admission. The lack of implementation cannot be explained by the discriminative ability, which was generally good. Model calibration was lacking in most studies, and therefore, it is difficult to judge whether a model, which performs well at group level, is also performing well for individual patients.

Future research should focus on validation, utility of additional predictors, exploration of electronic implementation in patient files to enable the clinical use of prediction models and analysis of their impact. Currently, impact analysis in prediction research is sparse, making it difficult to conclude whether a model is worth implementing as an adjunct to clinical evaluation. In the ED, it is worthwhile to investigate whether implementation of an admission prediction model reduces ED crowding and improves patient outcomes in terms of a shorter LOS at the ED and in the hospital.

Furthermore, we recommend that models should be validated and updated to judge generalisability to specific populations prior to implementation. We also recommend that with every external validation study, calibration should be reported.

## Conclusion

This systematic review identified 16 prognostic models for predicting admission in patients presenting to the ED. The models of Cameron and Lucke were well developed and have adequate predictive performance. We suggest that the effect of these models on ED LOS and crowding reduction should be examined, given that external validation and potentially updating of the models have taken place for the specific hospital ED.
