# Supplementary material for: Predicting inhospital admission at the emergency department: a systematic review
Source: Emerg Med J. 2021 Oct 28;39(3):191–8. doi: 10.1136/emermed-2020-210902 (PMC8921564; doi:10.1136/emermed-2020-210902)
Supplement: Supplementary data [file emermed-2020-210902supp001.pdf]

Appendix

Embase.com

('prediction'/exp OR 'predictive value'/exp OR 'predictive validity'/exp OR 'prediction and forecasting'/de OR 'risk factor'/de OR 'risk assessment'/de OR 'predictor variable'/exp OR (predict* OR ((risk OR hazard) NEAR/3 (factor* OR stratificat* OR assess*))):ab,ti) AND (hospitalization/exp OR 'hospital admission'/exp OR 'hospital readmission'/exp OR 'hospital discharge'/exp OR 'length of stay'/exp OR (hospitalizat* OR hospitalisat* OR rehospitalizat* OR rehospitalisat* OR (hospital NEAR/3 (admis* OR admit* OR readmis* OR readmit* OR discharg* OR stay)) OR ((length OR long* OR short* OR time OR Prolong*) NEAR/3 (stay* OR los))):ab,ti) AND ('emergency care'/exp OR 'emergency patient'/exp OR 'emergency ward'/exp OR 'emergency health service'/exp OR ((emergen* NEAR/3 (ward* OR department* OR patient* OR service* OR admiss* OR admit* OR hospital* OR call*))):ab,ti) AND ('cohort analysis'/exp OR 'follow up'/exp OR 'longitudinal study'/de OR 'retrospective study'/de OR 'prospective study'/de OR 'evaluation study'/de OR model/de OR 'disease model'/de OR 'population model'/de OR 'process model'/de OR simulation/exp OR algorithm/de OR 'validation process'/exp OR 'sensitivity and specificity'/exp OR 'scoring system'/exp OR 'decision tree'/de OR (model OR simulat* OR cohort* OR (follow* NEXT/1 up*) OR followup* OR longitudinal* OR retrospectiv* OR prospectiv* OR evaluation* OR algorithm* OR validat* OR sensitivit* OR specificit* OR score* OR (decision NEXT/1 tree*)):ab,ti) NOT ([Conference Abstract]/lim OR [Letter]/lim OR [Note]/lim OR [Editorial]/lim) AND [english]/lim NOT ((child/exp OR childhood/exp OR adolescent/exp OR adolescence/exp) NOT (adult/exp OR adulthood/exp)) NOT (pediatrics/exp OR (picu OR nicu OR picus OR nicus OR pediatric* OR paediatric*):ab,ti)

Medline ovid

("Predictive Value of Tests"/ OR "Forecasting"/ OR "Risk Factors"/ OR "risk assessment"/ OR (predict* OR ((risk OR hazard) ADJ3 (factor* OR stratificat* OR assess*))).ab,ti.) AND (exp hospitalization/ OR (hospitalizat* OR hospitalisat* OR rehospitalizat* OR rehospitalisat* OR (hospital ADJ3 (admis* OR admit* OR readmis* OR readmit* OR discharg*)) OR ((length OR long* OR short*) ADJ3 (stay* OR los))).ab,ti.) AND ("Emergency Medical Services"/ OR "emergencies"/ OR exp "Emergency Service, Hospital"/ OR ((emergen* ADJ3 (ward* OR department* OR patient* OR service* OR admiss* OR admit* OR hospital* OR call*))).ab,ti.) AND (exp "cohort studies"/ OR "evaluation study"/ OR exp "Models, Statistical"/ OR "Computer Simulation"/ OR "Models, Theoretical"/ OR Algorithms/ OR "Validation Studies"/ OR exp "sensitivity and specificity"/ OR "Decision Trees"/ OR (model OR simulat* OR cohort*

OR (follow* ADJ up*) OR followup* OR longitudinal* OR retrospectiv* OR prospectiv* OR evaluation* OR algorithm* OR validat* OR sensitivit* OR specificit* OR score* OR (decision ADJ tree*)).ab,ti.) NOT (letter OR news OR comment OR editorial OR congresses OR abstracts).pt. AND english.la. NOT ((exp child/ OR exp Infant/ OR adolescent/) NOT (exp adult/)) NOT (exp pediatrics/ OR (picu OR nicu OR picus OR nicus OR pediatric* OR paediatric*).ab,ti.)

Cochrane

((predict* OR ((risk OR hazard) NEAR/3 (factor* OR stratificat* OR assess*))):ab,ti) AND ((hospitalizat* OR hospitalisat* OR rehospitalizat* OR rehospitalisat* OR (hospital NEAR/3 (admis* OR admit* OR readmis* OR readmit* OR discharg*)) OR ((length OR long* OR short*) NEAR/3 (stay* OR los))):ab,ti) AND (((emergen* NEAR/3 (ward* OR department* OR patient* OR service* OR admiss* OR admit* OR hospital* OR call*))):ab,ti) AND ((model OR simulat* OR cohort* OR (follow* NEXT/1 up*) OR followup* OR longitudinal* OR retrospectiv* OR prospectiv* OR evaluation* OR algorithm* OR validat* OR sensitivit* OR specificit* OR score* OR (decision NEXT/1 tree*)):ab,ti)

Google scholar

prediction|"risk|hazard factor|stratification|assessment" hospitalization|rehospitalization|"hospital admission|discharge"|"length of stay" "emergency ward|department|patient|service" model|simulation|cohort|"follow up"|evaluation
