# Supplementary material for: Predicting inhospital admission at the emergency department: a systematic review
Source: Emerg Med J. 2021 Oct 28;39(3):191–8. doi: 10.1136/emermed-2020-210902 (PMC8921564; doi:10.1136/emermed-2020-210902)
Supplement: Supplementary data [file emermed-2020-210902supp003.pdf]

Potential bias	Items to be considered for potential bias	Cameron (2014)	Kraaijvanger	Lucke	Noel	Zlotnik
Participant selection	*Low risk of bias if:*					
	In- and exclusion criteria were adequately described	X	X	X	X	X
	Patient characteristics were adequately described		X	X	X	
	Study dates were noted	X	X	X	X	X
	Moderate risk of bias if:					
	Participant selection was described but not satisfying					
	High risk of bias if:					
	No adequate description of recruitment of study sample was given					
Predictor assessment	*Low risk of bias if:*					
	Predictor definitions were the same for all patients	x	x	x	x	x
	All predictors were available at the time of the model is intended to be used	x	x	x	x	x
	Predictors were measured with valid and reproducible methods such that misclassification was limited	x	x	x		x
	Handling of predictors in the modelling was described (e.g. continuous, categorized)	x	x	x	x	x
	Moderate risk of bias if:					
	One of the criteria was not satisfied				x	
	High risk of bias if:					
	Predictor assessment was not adequately described					
Outcome assessment	*Low risk of bias if:*					
	Definition of outcome was described adequately	x	x	x	x	x
	There was no loss of follow-up or <20%	x	x	x	x	x
	Moderate risk of bias if:					

	Loss of follow up was not described					
	High risk of bias if:					
	Measurement of outcome was unclear					
Model development	*Low risk of bias if:*					
	Number of outcomes in relation to number of candidate predictors (events per variable, EPV) was described and satisfying	x	x	x	x	x
	Number of participants with missing data was described				x	
	Compensation for missing data was performed	x		x		
	Methods used to select predictors for inclusion in multivariable analysis were described	x	x	x	x	x
	Selection of predictors during multivariable modelling was described	x	x	x		x
	Shrinkage methods were used to account for overfitting					
	Moderate risk of bias if:					
	The EPV was less than ten					
	Continuous predictors were categorized prior to inclusion in multivariable analysis	x (age)	x (age)	x	x (age)	x(age), number ED visits
	The methods for predictor selection were not described accurately					
	Participants with missing data were excluded				x	
	High risk of bias if:					
	Description of number of outcomes, missing data and predictor selection was lacking					
Analysis	*Low risk of bias if:*					
	Calibration was assessed and described	x	x	x	x	x
	Discrimination was determined	x	x	x	x	x
	No cut-off points were used	x	x	x	x	x
	External validation was performed, or internal validation accounted for optimism and shrinkage		x	x		

BMJ Publishing Group Limited (BMJ) disclaims all liability and responsibility arising from any reliance placed on this supplemental material which has been supplied by the author(s)

	Moderate risk of bias if:					
	Internal validation was performed without optimism correction	x				x
	External validation was performed in a population identical to the derivation cohort					
	Cut-off points were used					
	High risk of bias if:					
	No data about discrimination and calibration was described					
	No validation was performed				x	

Brink A, *et al. Emerg Med J* 2022; 39:191–198. doi: 10.1136/emermed-2020-210902
