# Supplementary material for: Predicting inhospital admission at the emergency department: a systematic review
Source: Emerg Med J. 2021 Oct 28;39(3):191–8. doi: 10.1136/emermed-2020-210902 (PMC8921564; doi:10.1136/emermed-2020-210902)
Supplement: Supplementary data [file emermed-2020-210902supp002.pdf]

Appendix B

Footnote belonging to Table 2

Demographics: *Age, sex, marital status, type of residence, insurance status.* *Vital signs:* *heart rate, systolic blood pressure, respiratory rate, oxygen saturation, AVPU, body temperature.* *Interventions:* *oxygen therapy, phlebotomised blood sample.* *Triage:* *ESI category, MTS category, patient acuity score.* *Previous care contacts:* *previous ED visit, previous hospital admission, hospitalized past six months, admitted less than one year ago, ED visit in previous thirty days, previous admission and discharge diagnosis.* *Chief complaint:* *chief complaint classification, complaint code, visit cause, MTS complaint code.* *Drug use:* *six or more medications, polypharmacy, number of drugs in previous three months.* *Mobility and dependency:* *difficulties with walking, difficulties with transfer, recent falls, need for help, vision problems, memory problems, more help since hospitalisation.* *ED entrance:* *referred by GP, arrived in ambulance, mode of arrival, referral, visit source.* *Professional assessment:* *triage nurse prediction, professional recommendation, type of specialist, VAS score.*
